# Metabolic changes assessed by 1H MR spectroscopy in the corpus callosum of post-COVID patients

**DOI:** 10.1007/s10334-024-01171-w

**Published:** 2024-06-12

**Authors:** Dita Pajuelo, Monika Dezortova, Milan Hajek, Marketa Ibrahimova, Ibrahim Ibrahim

**Affiliations:** 1 Institute for Clinical and Experimental Medicine, Department of Diagnostic and Interventional Radiology, Videnska 1958/9, 140 21 PRAGUE 4, Prague, Czech Republic; 2 Laboratory of Immunology, Thomayer University Hospital, Prague, Czech Republic

**Keywords:** Proton MR spectroscopy, COVID-19, The splenium of the corpus callosum, Metabolism

## Abstract

**Objective:**

Many patients with long COVID experience neurological and psychological symptoms. Signal abnormalities on MR images in the corpus callosum have been reported. Knowledge about the metabolic profile in the splenium of the corpus callosum (CCS) may contribute to a better understanding of the pathophysiology of long COVID.

**Materials and methods:**

Eighty-one subjects underwent proton MR spectroscopy examination. The metabolic concentrations of total N-acetylaspartate (NAA), choline-containing compounds (Cho), total creatine (Cr), myo-inositol (mI), and NAA/Cho in the CCS were statistically compared in the group of patients containing 58 subjects with positive IgG COVID-19 antibodies or positive SARS-CoV-2 qPCR test at least two months before the MR and the group of healthy controls containing 23 subjects with negative IgG antibodies.

**Results:**

An age-dependent effect of SARS-CoV-2 on Cho concentrations in the CCS has been observed. Considering the subjective threshold of age = 40 years, older patients showed significantly increased Cho concentrations in the CCS than older healthy controls (*p* = 0.02). NAA, Cr, and mI were unchanged. All metabolite concentrations in the CCS of younger post-COVID-19 patients remained unaffected by SARS-CoV-2. Cho did not show any difference between symptomatic and asymptomatic patients (*p* = 0.91).

**Discussion:**

Our results suggest that SARS-CoV-2 disproportionately increases Cho concentration in the CCS among older post-COVID-19 patients compared to younger ones. The observed changes in Cho may be related to the microstructural reorganization in the CCS also reported in diffusion measurements rather than increased membrane turnover. These changes do not seem to be related to neuropsychological problems of the post-COVID-19 patients. Further metabolic studies are recommended to confirm these observations.

## Introduction

Coronavirus disease 2019 (COVID-19) is an infectious disease caused by the severe acute respiratory syndrome coronavirus 2 (SARS-CoV-2) [1]. Patients with COVID-19 may present a variety of symptoms from asymptomatic infections, mild symptoms such as fever, cough, and shortness of breath, to severe life-threatening pneumonia and multiorgan involvement [2, 3]. SARS-CoV-2 also affects the central nervous system (CNS), resulting in neurological symptoms such as loss of smell and taste [4]. A substantial proportion of affected patients do not fully recover from COVID-19, going on to develop long COVID (a postviral syndrome) [5]. The patients experience neurological and psychological symptoms associated with the disease, including headaches, brain inflammation, fatigue, muscle pain, depression, and anxiety [6, 7].

Systematic reviews and MR brain studies report parenchymal brain abnormalities, subcortical micro- and macro-bleeds, cortico-subcortical swelling, and non-specific deep white matter changes visible on MR images [2, 8]. Signal and diffusion abnormalities are extended predominantly to the corpus callosum, cingulate cortex, and insula, jointly implicating the olfactory brain network [2, 9]. Sawlani et al. also reported white matter abnormalities especially involving the splenium of the corpus callosum and brainstem in COVID-19 patients with a range of medical and neurological comorbidities [10].

Corona viruses can invade the CNS by two mechanisms: viral replication into glial or neuronal cells of the brain or autoimmune reaction [11, 12]. Autopsy studies of patients with COVID-19 show prominent neuroinflammation and activated microglia [13, 14]. Glial and neuronal metabolic abnormalities in post-COVID-19 patients may be non-invasively assessed by proton magnetic resonance spectroscopy (1H MRS). N-acetylaspartate and N-acetylaspartylglutamate (NAA) are considered to be markers of neuronal viability and density [15, 16]. Neuroinflammation is typically represented by elevated glial marker myo-inositol (mI), concomitantly elevated choline-containing compounds (Cho), and total creatine [17], due to their higher concentrations in glia than in neurons [14]. Creatine together with phosphocreatine, referred to as total creatine (Cr), is considered as a marker of cerebral bioenergetics. Choline-containing compounds comprising mainly phosphocholine, glycerophosphocholine, and free choline reflect cell membrane constituents and membrane turnover [16]. Myo-inositol is considered as a glial cell marker, it is connected to the osmoregulation of astrocytes, and it is important for the integrity of the cells. Increased mI probably reflects a glial activation and proliferation; however, its exact function remains unclear [18].

Post-COVID-19 patients with persistent neuropsychiatric symptoms revealed in the anterior cingulate cortex lower NAA indicating neuronal injury and lower mI reflecting glial dysfunction, possibly related to mitochondrial dysfunction and oxidative stress [14]. Their subset of non-hospitalized group showed decreased mI compared to hospitalized post-COVID-19 patients. The non-hospitalized group further showed significantly decreased mI, Cho, Cr, and Glx in frontal white matter (WM) compared to healthy controls. Reda et al. [12] reported decreased NAA and NAA/Cr and elevated Cho, Glx, and lactate (Lac) in non-hemorrhagic lesions, increased Lac/Cr in necrotizing leukoencephalopathy, and no changes in apparently normal WM in patients with acute COVID-19 infection with neurological symptoms.

COVID-19-associated necrotizing leukoencephalopathy showed NAA/Cr reduction and elevation of Cho/Cr, Glx/Cr, Lac/Cr signal ratios, and Lac [12, 19]. Similar metabolic patterns, but with less pronounced changes and without an increased Lac/Cr ratio, were also seen in patients with COVID-19 postcardiac arrest and in non-COVID-19 patients with delayed posthypoxic leukoencephalopathy [19]. Sklinda et al. did not found any changes in values of NAA, Cho, and Cr in patients hospitalized due to symptoms of severe brain fog (i.e., insomnia, sudden impairment of cognitive function, headache, and depression) [20]. Lin et al. [21] reported increased Cho in the cingulate gyrus, which may be reflective of neuroinflammatory changes due to long COVID.

Although many MRI studies report signal abnormalities on MR images in the corpus callosum [2, 9, 10], to our knowledge, no MRS study evaluating metabolic changes in the splenium of the corpus callosum (CCS) has been performed yet. The corpus callosum forms a bridge between the cerebral hemispheres, containing crossing axonal fibers from both hemispheres [22]. Its primary role lies in facilitating interhemispheric collaboration [23]. The CCS is the most posterior part of the corpus callosum [22] and its lesions may result in the disconnection of the cerebral hemispheres, with disruption of higher cortical function, loss of conscious processes, and delirious behavior [24]. As many post-COVID-19 patients experience neuropsychological symptoms [5–7, 14], knowledge about possible changes in the metabolic profile in the CCS may contribute to a better understanding of the pathophysiology of long COVID.

## Methods

### Subjects

Eighty-four subjects (mean age: 42.8 ± 12.5, 52 females/32 males) were included in this study and underwent a 1H MRS examination. Thirty-six subjects (symptPAT) were recruited by a neurologist based on persistent post-COVID-19 neuropsychological symptoms (i.e., fatigue, concentration and memory problems, headaches, insomnia, psychological problems, etc.) for at least two months following a history of COVID-19 infection (positive qPCR test). The remaining 48 subjects had never tested positive by qPCR before and were, therefore, tested for the presence of COVID-19 antibodies, primarily IgG antibodies in the blood (serology test) to detect evidence of prior infection. These subjects were subsequently divided to the asymptomatic patients or healthy controls according to IgG results (for details, see the Results/Classification of subjects section). In addition, the subjects also completed a health questionnaire. The exclusion criteria were the age up to 70 years and a history of neurological and mental disorder other than post-COVID-19 syndrome. The data were collected between March 2022 and March 2023.

All the participants were informed about the purpose of the study and signed a written consent prior to examination. This study conformed to the ethical guidelines of the 1975 Declaration of Helsinki and was approved by the Joint Ethics Committee of the Institute for Clinical and Experimental Medicine and Thomayer University Hospital, Prague, Czech Republic (No. 29451/21; G-21–70).

### MR examination

All the subjects underwent an MR examination on a 3 T Magnetom VIDA scanner (Siemens Medical Systems, Erlangen, Germany) equipped with a 64-channel volume head/neck coil. The MRI part comprised a standard clinical MRI protocol including T1-weighted sagittal images obtained using a three-dimensional (3D) magnetization-prepared rapid gradient-echo (MPRAGE) sequence (echo time (TE)/repetition time (TR)/inversion time (TI)/number of acquisitions (NA) = 2.25 ms/2000 ms/900 ms/ 1, iPAT = 3, resolution 0.9 × 0.9 × 0.9 mm) used to calculate the tissue proportion in the MRS volume of interest (VOI). The MR images were visually assessed by neuroradiologists to preclude the presence of lesions and used for VOI localization. Three subjects were excluded from the study due to abnormal MRI findings (subdural cyst and hematoma, abnormal gliosis, low grade glioma). These findings were incidental, and the patients were unaware of them.

**Fig. 1:**
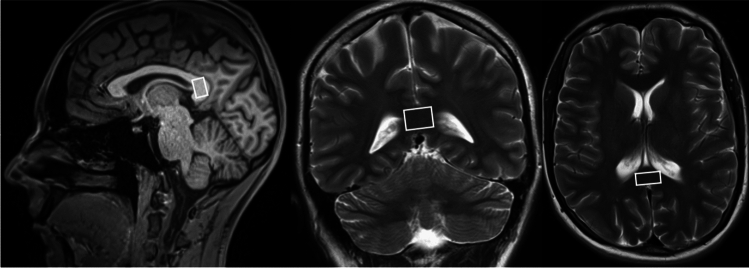
An example of the spectroscopic voxel position in the splenium of the corpus callosum on a sagittal T1-weighted image and on coronal and transversal T2-weighted MR images. The figure was created in Corel Graphics Suite

The 1H MRS protocol included a single voxel spectroscopy (SVS) measurement from the splenium of the corpus callosum (CCS). Spectra were obtained using the Point Resolved Spectroscopy (PRESS) sequence: TE/TR/NA = 30 ms/5000 ms/96 with (and 30 ms/5000 ms/1 without) water suppression, a default nominal voxel volume of 1.4 ml, and acquisition time 8 min 25 s. The VOI (default 8 × 15 × 12 mm^3^) was positioned in the CCS individually to minimize the contamination of cerebrospinal fluid (CSF) (Fig. 1). Spectra were evaluated using LCModel software [25]. A simulated basis set with prior knowledge for 25 metabolites was used (detailed information about the metabolites including the chemical shifts and multiplicity may be found in the caption of Fig. 7 in [16]; lipid signals contained prior knowledge for Lip at 1.3, 0.9, and 2.0 ppm. Prior knowledge for macromolecules at 0.9, 1.2, 1.4, 1.7, and 2.0 ppm were added). A water signal was used as an internal standard for the calculation of the metabolic concentrations. NAA, Cr, Cho, mI concentrations, and the NAA/Cho ratio were evaluated and corrected for water content [26] (equation No. 8). This procedure requires information about the proportion of gray matter, white matter, and cerebrospinal fluid in each VOI [26] (equation Nos. 5–7), which was obtained by MPRAGE image segmentation using an SPM8 program [27] and an in-house Java-based tool for obtaining average tissue values from segmentation maps in the region of the examined VOI. Corrections for relaxation times were not made because their effect is small (< 4%) when using short TE and sufficiently long TR [28] and it was not necessary for the purposes of this study.

**Fig. 2:**
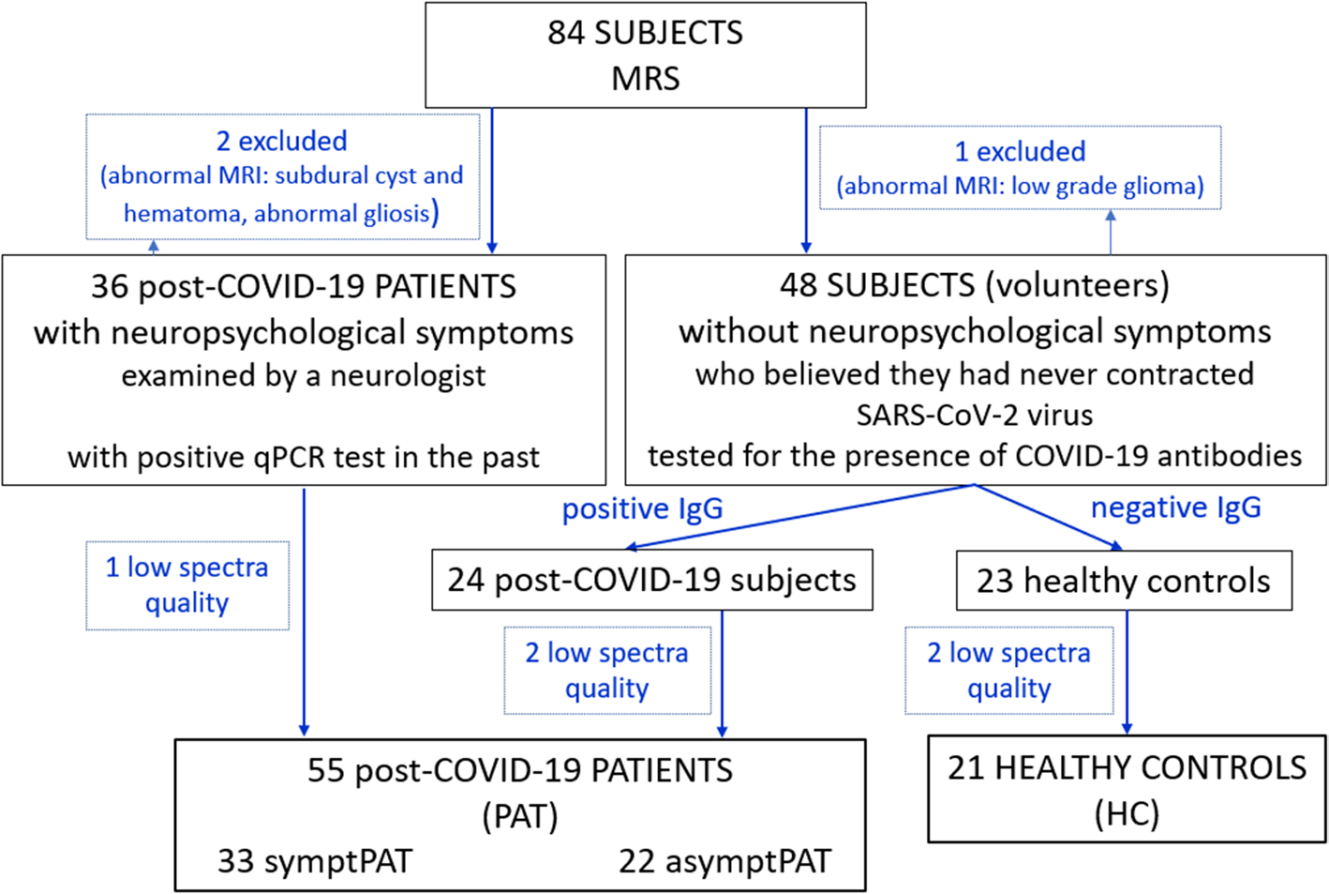
Inclusion graph. MRS: magnetic resonance spectroscopy; PAT: subjects with positive SARS-CoV-2 qPCR test at least two months before the MR examination or positive COVID-19 IgG antibodies (> 22 kU/l); HC: subjects with COVID-19 IgG < 22 kU/l; symptPAT: patients with neuropsychological symptoms; asymptPAT: asymptomatic patients. The figure was created in Corel Graphics Suite

Only spectra without visible spectral artifacts with a signal-to-noise ratio (SNR) > 4 and half-width at half maximum (FWHM) of the water signal below 0.08 ppm were included in the further analysis (76 subjects, Fig. 2) to ensure reliable metabolite quantification. Five subjects were excluded for that reason.

### Antibody tests

The subjects included in this study were tested for COVID-19 antibodies. The specific antibodies to the nucleocapsid of SARS-CoV-2 were detected using chemiluminescence immunoassay (TestLine Clinical Diagnostics) on a KleeYa analyzer (Stratec). The determination was performed for antibodies in the IgM and IgA classes, indicating the acute phase of infection, and in the IgG class, where the antibodies are anamnestic [29]. Antibody levels higher than 22 kU/l were interpreted as positive.

### Statistical analysis

The Shapiro–Wilk normality test was used for verification of the normal (Gaussian) data distribution. The Pearson correlation was used to test the relationships between metabolic concentrations and age. Differences between groups were assessed by ANCOVA with separate slopes and with two factors (age and group) and their interaction. The analysis by ANCOVA was run twice: firstly, with two groups (patients and healthy controls); secondly, with three groups (symptomatic patients, asymptomatic patients and healthy controls). Tukey’s HSD test was employed for all pairwise comparisons in the case of three groups. Furthermore, the Holm correction for multiple comparisons was applied in case of the correlation analysis, 2- and 3-group ANCOVA, and the comparison of older and younger subjects. A *p*-value < 0.05 was considered as statistically significant. The statistical analyses were performed using the JMP software (https://www.jmp.com/en_us/home.html) and the GraphPad Prism program, version 10.1.

## Results

### Classification of subjects

The COVID-19 antibody tests revealed that 24 out of 47 subjects (51%) were tested positive for IgG antibodies from previous COVID-19 infection (Fig. 2). Another thirty-four symptomatic patients had positive SARS-CoV-2 qPCR test at least two months before the MR examination. All these subjects were designated as patients (PAT). The final PAT group contained 55 subjects (three patients were excluded because of low spectra quality). Twenty-two patients (asymptPAT, mean age: 45.0 ± 11.7, 14 females/8 males, IgG = 186 ± 118 kU/l, IgM = 14 ± 5 kU/l, IgA < the detection capability of the method 5 kU/l in 18 patients, in the rest 5 patients IgA = 13 ± 8 kU/l) were asymptomatic without any post-COVID-19 neuropsychological symptoms (according to an anamnestic questionnaire). Thirty-three patients (symptPAT, mean age: 43.3 ± 12.8, 23 females/10 males, IgG, IgM, IgA data not available) were examined by a neurologist after contraction of SARS-CoV-2 and they had experienced persistent neurological symptoms for at least two months following a history of COVID-19 infection: fatigue (76%), concentration and memory problems (67%), headaches (73%), muscle and joint pain (64%), cough (48%), loss of taste and smell (52%), hospitalization (15%), insomnia (48%), and psychological problems (21%). Out of the 23 subjects with a negative COVID-19 IgG test, who were designated as the healthy controls (HC), two subjects were excluded because of low spectra quality. None of the final HC (mean age: 39.8 ± 12.8, 11 females/10 males, IgG = 9 ± 3 kU/l, IgM = 11 ± 3 kU/l, IgA < the detection capability of the method 5 kU/l in 20 patients, in 1 patient IgA = 8.8 kU/l) had any history of neurological or mental disorders.

There was no statistically significant difference between the PAT and HC groups in age.

### MRS findings

**Fig. 3:**
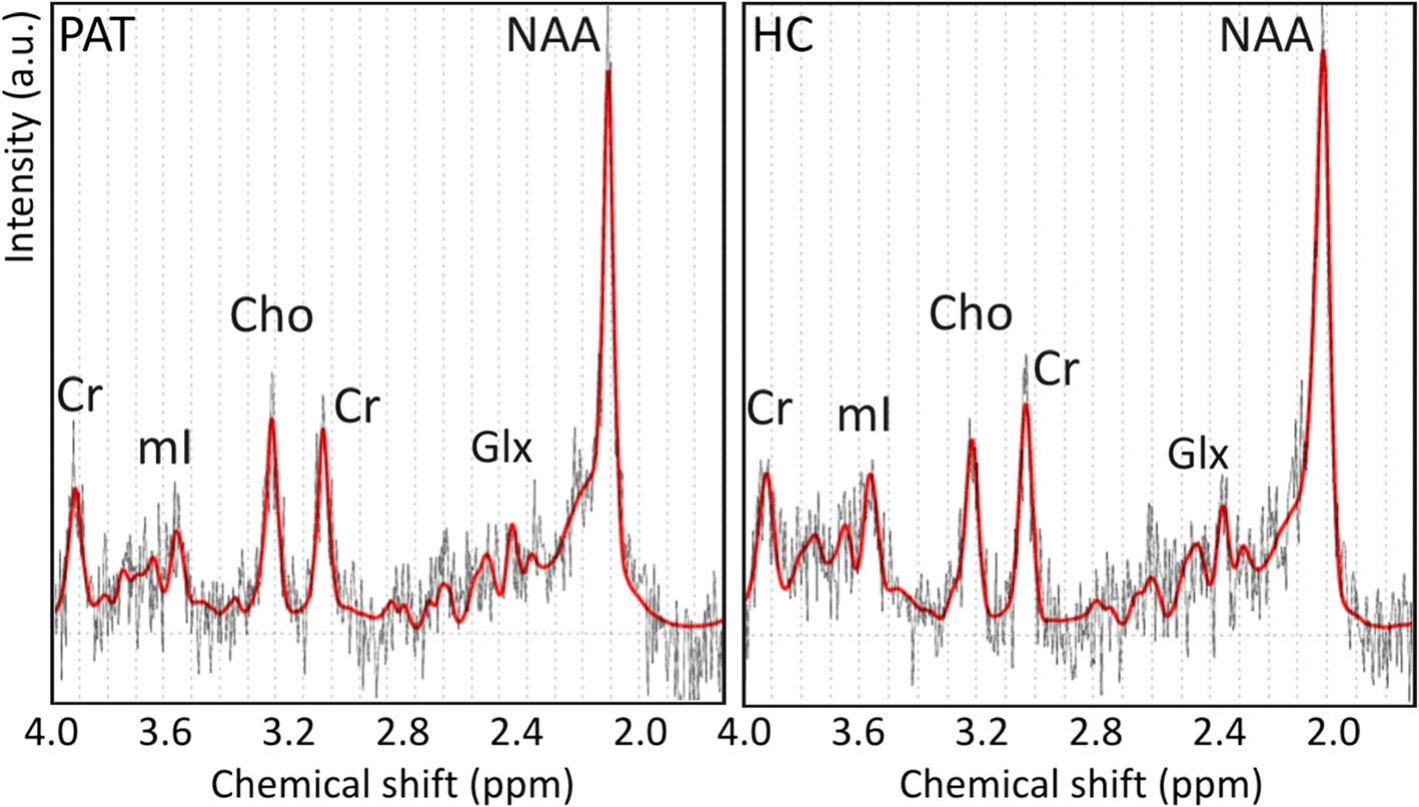
An example of 1H MR spectrum measured using the PRESS sequence with short echo time from the splenium of the corpus callosum in a symptomatic post-COVID-19 patient and a healthy control. The figure was created in Corel Graphics Suite. a.u.: arbitrary unit; ppm: parts per million; PAT: subjects with COVID-19 IgG antibodies > 22 kU/l or with positive SARS-CoV-2 PCR test at least two months before the MR examination; HC: subjects with COVID-19 IgG < 22 kU/l; Cr: total creatine; mI: myo-inositol; Cho: choline-containing compounds; Glx: glutamine + glutamate; NAA: total N-acetylaspartate

The distribution of GM, WM, and CSF in the VOI calculated in the PAT and HC groups did not show any significant differences (Table 1). The mean size of VOI in both PAT and HC group was 1.4 ± 0.1 ml. The SNR of spectra and Cramér-Rao Lower Bounds (CRLB) of the spectral fit for each metabolite did not significantly differ between the PAT and HC groups (Table 2). Examples of 1H MRS spectrum in CCS in a patient and in a healthy control are shown in Fig. 3.

**Table 1:** Mean distribution of WM, GM, and CSF in the VOI and their standard deviations

	WM [%]	GM [%]	CSF [%]
PAT group	98.0 ± 2.5	1.6 ± 1.9	0.4 ± 0.8
HC group	97.1 ± 2.4	2.2 ± 1.8	0.5 ± 0.8

*WM* white matter; *GM* gray matter; *CSF* cerebrospinal fluid, *VOI* volume of interest; *PAT* group of 55 subjects with positive IgG antibodies to the nucleocapsid of SARS-CoV-2 or with positive SARS-CoV-2 qPCR test at least two months before the MR examination; *HC* 21 healthy controls with COVID-19 IgG < 22 kU/l

**Table 2:** The mean SNR of the spectra and the mean Cramér-Rao Lower Bounds of the spectral fit for each metabolite and their standard deviations in the PAT and HC groups

	SNR	CRLB [%] NAA	CRLB [%] Cr	CRLB [%] Cho	CRLB [%] mI
PAT group	7 ± 1	5.6 ± 0.7	9.6 ± 1.5	10.4 ± 2.1	13.4 ± 3.5
HC group	7 ± 1	5.2 ± 0.9	9.4 ± 1.2	10.8 ± 1.8	13.5 ± 3.2

*PAT*: subjects with COVID-19 IgG antibodies > 22 kU/l or with positive SARS-CoV-2 qPCR test at least two months before the MR examination; *HC*: subjects with COVID-19 IgG < 22 kU/lmean; *SNR*: signal-to-noise ratio; *CRLB*: Cramér-Rao Lower Bounds of the spectral fit, *NAA*: total N-acetylaspartate; *Cr*: total creatine; *Cho*: choline-containing compounds; *mI*: myo-inositol

**Fig. 4:**
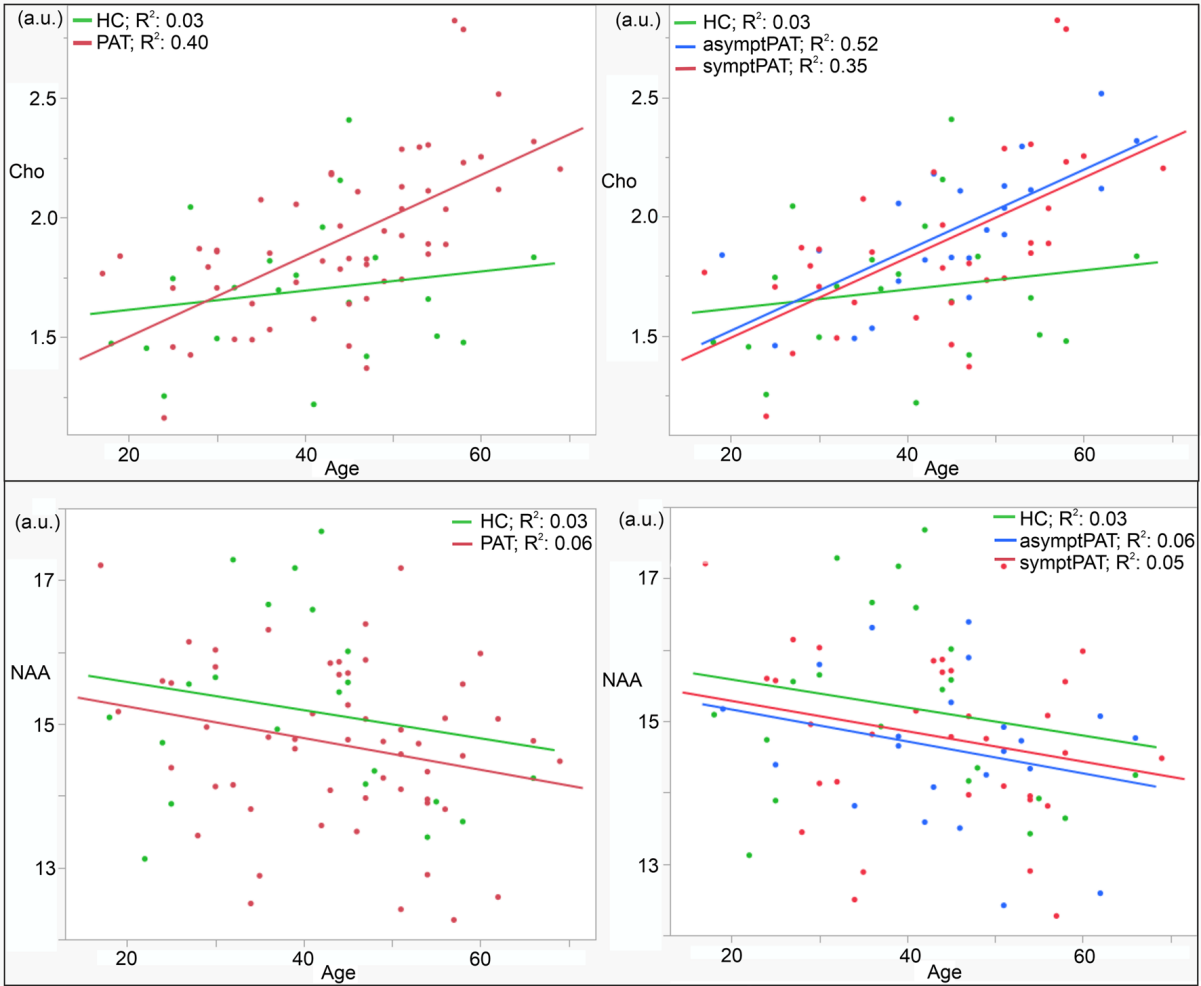
The linear regression plots of age and the individual metabolic concentrations for the PAT and HC groups. PAT: subjects with COVID-19 IgG antibodies > 22 kU/l or with positive SARS-CoV-2 qPCR test at least two months before the MR examination; symptPAT: patients with neuropsychological symptoms; asymptPAT: asymptomatic patients; HC: subjects with COVID-19 IgG < 22 kU/l; NAA: total N-acetylaspartate; Cho: choline-containing compounds

All evaluated metabolic data had normal distribution (Shapiro–Wilk normality test: PAT: *p*-value: NAA: 0.63, Cr: 0.63, Cho: 0.28, Ins: 0.06, NAA/Cho: 0.19; HC: NAA: 0.51, Cr: 0.30, Cho: 0.62, Ins: 0.88, NAA/Cho: 0.21). The Pearson correlation showed statistically significant linear relationships between age and all metabolic concentrations (NAA: *r* = −0.24, *p* = 0.04; Cr: *r* = 0.34, *p* = 0.006; Cho: *r* = 0.53, *p* < 0.0001; Ins: *r* = 0.42, *p* = 0.0006; NAA/Cho: *r* = −0.54, *p* < 0.0001) in all the subjects. Therefore, ANCOVA with the age as a covariate was used for the comparison of the PAT and HC groups. The analysis revealed that Cho adjusted for age was significantly increased and NAA/Cho adjusted for age was significantly decreased in the CCS in the PAT group compared to the HC group (Table 3). However, Cho showed a significant interaction between *group* and *age*. Therefore, the linear regression plots of age and Cho as well as the NAA concentrations for the PAT and HC groups and for the symptPAT, asymptPAT, and HC groups are shown in Fig. 4. When considering Cho-age dependence and dividing the PAT and HC groups into younger (< 40 years old) and older (> 40 years old) subgroups based on a subjective threshold of 40 years of age, Cho was found to be significantly increased only in the older PAT group compared to the older HC group (*p* = 0.02), and correspondingly, NAA/Cho was significantly decreased in older PAT compared to older HC (*p* = 0.01). No significant difference in Cho and NAA/Cho values between the younger PAT and younger HC groups was found (*p* = 0.54; 0.72 resp.).

**Table 3:** Metabolic concentrations and NAA/Cho ratio in the splenium of the corpus callosum in the post-COVID-19 patients and healthy controls. Least Squares Means adjusted for age ± their Standard Errors are presented

Concentrations	NAA [a.u.]	Cr [a.u.]	Cho [a.u.]	mI [a.u.]	NAA/Cho
PAT (*n* = 55)	14.74 ± 0.16	5.54 ± 0.09	1.89 ± 0.04*	6.73 ± 0.20	8.06 ± 0.19*
HC (*n* = 21)	15.14 ± 0.27	5.42 ± 0.14	1.71 ± 0.06*	6.54 ± 0.34	9.08 ± 0.31*
Effect group test: *p*	0.42	0.99	0.02*	0.63	0.01*
Effect age test: *p*	0.19	0.11	0.0008*	0.01*	0.0002*
Effect group*age test: *p*	0.92	0.14	0.047*	0.42	0.21

*PAT*: subjects with COVID-19 IgG antibodies > 22 kU/l or with positive SARS-CoV-2 qPCR test at least two months before the MR examination; *HC*: subjects with COVID-19 IgG < 22 kU/l; *NAA*: total N-acetylaspartate; *Cr*: total creatine; *Cho*: choline-containing compounds; *mI*: myo-inositol; a.u.: arbitrary unit; *p*: value of ANCOVA statistics with the age as a covariate corrected for multiple comparison; *p < 0.05

No statistically significant differences were observed in the case of NAA, Cr, or mI. Lactate was negligible in both groups, and Glx was not evaluated due to low SNR. No correlation between metabolite levels and IgG has been found (slopes ranged between −0.002 and 0.002).

**Fig. 5:**
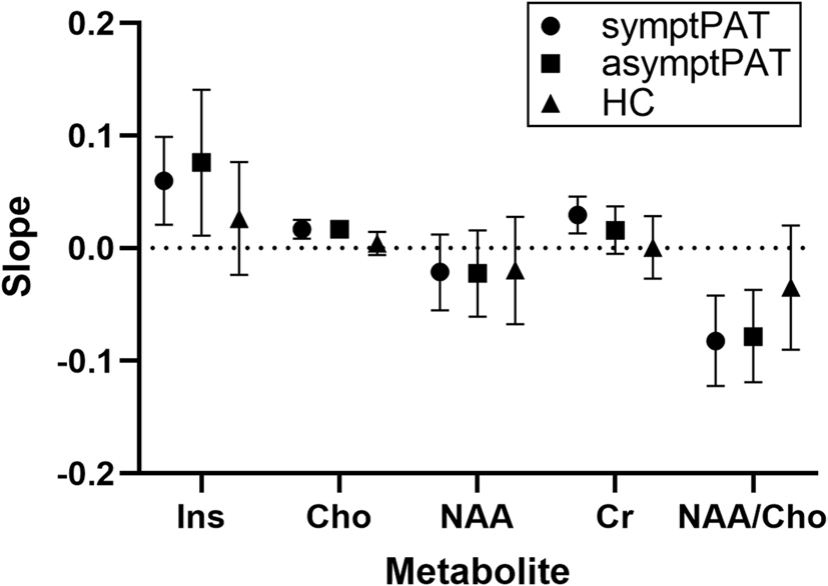
Slopes of the linear regression fit of metabolic concentrations – age dependence with their 95% confidence intervals for the symptPAT, asymptPAT, and HC groups. PAT: subjects with COVID-19 IgG antibodies > 22 kU/l or with positive SARS-CoV-2 qPCR test at least 2 months before MR examination; symptPAT: patients with neuropsychological symptoms; asymptPAT: asymptomatic patients; HC: subjects with COVID-19 IgG < 22 kU/l; mI: myo-inositol; Cho: choline-containing compounds; NAA: total N-acetylaspartate; Cr: total creatine; NAA/Cho: NAA/Cho ratio

The ANCOVA comparison between symptPAT, asymptPAT, and HC revealed that none of the metabolites showed significant interaction between *group* and *age* (effect group*age test: NAA, Cr, Cho, mI, NAA/Cho: *p* = 0.99, 0.14, 0.08, 0.42, 0.29). The analysis found statistically significant differences between groups only in the case of Cho and NAA/Cho (Cho: *p* = 0.04; NAA/Cho: *p* = 0.02). For Cho, however, pairwise comparisons did not show any difference between symptPAT and asymptPAT (*p* = 0.91), symptPAT and HC (*p* = 0.08) nor asymptPAT and HC (*p* = 0.05). For NAA/Cho, no significant differences were found between symptPAT and asymptPAT (*p* = 0.67) nor symptPAT and HC (*p* = 0.07). AsymptPAT showed a statistically significant decrease in NAA/Cho compared to HC (*p* = 0.02). The comparison of slopes of the linear regression fit of metabolic concentrations on age for the symptPAT, asymptPAT, and HC groups is presented in Fig. 5.

## Discussion

The majority of studies dealing with patients after suffering from COVID-19 with neuropsychiatric symptoms commonly report microstructural changes in the brain, predominantly in the corpus callosum (anterior part as well as splenium), manifesting as signal and diffusion abnormalities [2, 7, 9, 10]. Although the changes in the microstructure may also imply metabolic abnormalities, to our knowledge, to-date, metabolism using MRS has been studied only in the anterior cingulate cortex, frontal WM, and COVID-19-related lesions visible on MRI [12, 14, 19–21, 30, 31]. For this reason, our study focused specifically on the metabolism in the splenium of the corpus callosum.

Our study revealed an age-dependent effect of SARS-CoV-2 on Cho concentrations in the splenium of the corpus callosum (see Fig. 4). While all metabolic concentrations showed significant linear dependence on age, as indicated by Pearson’s r, only Cho in CCS exhibited a significantly higher slope in the post-COVID-19 patient group compared to the healthy control group. This suggests that SARS-CoV-2 disproportionately increases Cho concentration in the CCS among older post-COVID-19 patients compared to younger ones. Our analysis demonstrated that Cho concentrations, adjusted for age in the CCS, differed significantly between all PAT and HC groups. However, considering a subjective threshold of 40 years of age, Cho was significantly increased only in the older PAT group compared to the older HC group. Our results are consistent with the findings of other studies showing a higher vulnerability of older populations to COVID-19 than younger adults [32]. Due to the strong effect of SARS-CoV-2 on Cho-age dependence and no effect on NAA-age dependence, NAA/Cho was also significantly more decreased in older patients than in older healthy subjects. NAA-age dependence in the PAT and HC groups were similar (see Fig. 4). The concentrations of NAA, mI, and Cr were stable. Interestingly, the PAT group did not reveal lower NAA indicating neuronal injury nor lower mI reflecting glial dysfunction in the CCS as post-COVID-19 patients with persistent neuropsychiatric symptoms in the anterior cingulate cortex [14]. It is possible to speculate that increased choline-containing compounds may be attributed to the microstructural changes in the CCS. Unfortunately, MRS relaxometry was not included in the examination protocol. Several studies previously reported lesions in the CCS with restricted diffusion and low values of apparent diffusion coefficient in hospitalized patients [9, 10]. Ibrahim et al. found increased values of mean diffusivity (MD) and decreased fractional anisotropy in post-COVID-19 patients in the forceps major without apparent signal abnormalities on standard MR images [7]. Diffusion abnormalities may show membrane reorganization of the axonal fibers between both hemispheres. As the MR visible Cho signal only reflects water-soluble choline metabolites such as phosphocholine and glycerophosphocholine, relatively immobile membrane components as sphingomyelin and phosphatidylcholine do not contribute to the total Cho signal intensity in the healthy tissue [16]. However, in the case of microstructural changes in the tissue, it is possible to speculate that changes in the arrangement of cell membranes may occur and tightly bound molecules may be released, become visible using MR spectroscopy, and cause an increase in the choline signal. As other metabolites are stable in the CCS, this explanation is more probable than neuroinflammation, glial dysfunction, or increased membrane turnover reported in other brain regions [12, 14, 19, 21].

Interestingly, 22 subjects with positive IgG antibodies to the nucleocapsid of SARS-CoV-2 believed they had never contracted COVID-19 (according to an anamnestic questionnaire) indicating they had passed COVID-19 infection completely asymptomatically without any post-COVID-19 neuropsychological symptoms. Simultaneously, the low levels of IgM and IgA classes indicated a non-acute phase of infection. Although these patients were not examined by a neurologist, and information about their health status during the COVID-19 pandemic is only subjective, we divided the PAT group into symptomatic and asymptomatic. At the same time, we would like to add that we believe that dividing subjects into PAT and HC groups based on IgG antibody levels or a positive qPCR in the past is more objective than the subjective division into symptPAT, asymptPAT, and HC. AsymptPAT revealed similar metabolic patterns to symptPAT (Fig. 4, ). Surprisingly, a subgroup of asymptomatic patients also revealed an age-dependent increase of choline-containing compounds in the CCS. Increased Cho was found in both symptomatic and asymptomatic older patients compared to healthy older controls. These results imply that the increased Cho in the CCS should not be related to neuropsychological symptoms. Although lesions in the CCS may result in neuropsychiatric problems [24], none of the subjects in our study were found to have lesions in the CCS.

In this study, we also evaluated the NAA/Cho ratio. This ratio is often used in metabolic studies and routine clinical practice as a parameter indicating metabolic changes in damaged brain tissue. Its calculation does not require special software or corrections as in the case of the calculation of individual metabolite concentrations. Although this is not a specific marker or characteristic only for COVID-19, a significantly decreased NAA/Cho ratio in older patients (driven by increased Cho in older patients) shows that it is the appropriate parameter for evaluating metabolic changes in the CCS in patients after COVID-19 infection even using a routine clinical protocol containing 1H MRS.

The present study has several limitations. Mainly, low SNR related to small VOI (1.4 ml). It would be desirable to have a minimum SNR = 10 which is important for accurate and precise metabolite quantification. However, only slight variations in absolute metabolite concentrations with SNR in the case of singlet signals in clinical conditions have been shown [33–35]. Increasing the number of acquisitions by a factor of 2 would increase the measurement time by a factor of 2 (more than 16 min only for one spectrum resulting in the risk of patient movement), but would result only in a 1.4-fold change in SNR (from mean SNR = 7 to 10). Narrow peaks (good B_0_ shimming), the efficiency of water suppression, and the elimination of unwanted coherences using a smaller voxel volume are more important than the SNR [33, 34, 36]. As the CCS is a small structure, small VOI dimensions were used to prevent contamination of metabolic signals from adjacent brain structures and CSF and to prevent loss of specificity. Low SNR is problematic in the case of multiplets such as Glx with more sophisticated line shapes. Therefore, these metabolites were not evaluated, although some MRS studies reported changes in Glx values in the frontal gray and white matter in long COVID patients [15, 21, 22, 24]. This study focused on an accurate quantification for precise detection of even small changes in NAA, Cr, Cho, and mI. Secondly, the data were acquired only from the CCS using a 3 T MR tomograph. In future studies, it is advisable to perform the measurement at 7 T to improve the sensitivity of MRS in small regions of interest and to also study other brain regions such as frontal white and gray matter. Thirdly, a methodological weakness is a variable of the subject’s symptoms, their severity, and duration, and therefore, many more subjects are needed. However, in our case, this seems to be unimportant as the changes in Cho concentrations and NAA/Cho were found even in asymptomatic post-COVID-19 subjects. Finally, it is also possible to see a limitation in missing information regarding relaxation times. In this study, it was not necessary to calculate T1 and T2 relaxation times due to the application of a short TE together with a long TR in the MRS sequence. However, relaxometry may help create an idea of the extracellular space geometry. Despite these limitations, MR spectroscopy remains the most suitable non-invasive method for evaluating in vivo metabolic changes in the brain tissue.

## Conclusion

An age-dependent effect of SARS-CoV-2 on concentrations of choline-containing compounds in the splenium of the corpus callosum has been found. Significantly increased Cho concentrations and decreased NAA/Cho were found in the CCS in the older post-COVID-19 subjects. Metabolism in the CCS of younger post-COVID-19 patients seems to be unaffected by SARS-CoV-2. The patients revealed similar metabolic patterns regardless of the severity of the symptoms. These changes should therefore not be related to neuropsychological problems of the post-COVID-19 patients. The observed changes in choline-containing compounds may be related to the microstructural reorganization in the corpus callosum (also reported in diffusion measurements) rather than increased membrane turnover. The CCS revealed a normal neuronal function, normal bioenergetic and glial metabolism in post-COVID-19 patients, as concentrations of total N-acetylaspartate, total creatine, and myo-inositol remained stable. Nevertheless, further metabolic studies are recommended to confirm these observations.

## Data Availability

The data that support the findings of this study are available from the corresponding author upon reasonable request.
